# **COVID-19, Trade, and Health: This Changes Everything?** Comment on "What Generates Attention to Health in Trade Policy-Making? Lessons From Success in Tobacco Control and Access to Medicines: A Qualitative Study of Australia and the (Comprehensive and Progressive) Trans-Pacific Partnership"

**DOI:** 10.34172/ijhpm.2020.220

**Published:** 2020-11-16

**Authors:** Pepita Barlow

**Affiliations:** Department of Health Policy, London School of Economics and Political Science, London, UK.

**Keywords:** Trade Liberalisation, United Kingdom, Nutrition, Political Economy of Health

## Abstract

Townsend and colleagues highlighted the myriad political forces which fostered attention to health issues during negotiations to establish a new trans-pacific trade deal in Australia (the CP-TPP [Comprehensive and Progressive Agreement for Trans-Pacific Partnership], formerly known as TPP). Among the factors they identify, exporter interests and exogenous events helped to generate attention to trade-related concerns about tobacco and access medicines, and limited attention to nutrition and alcohol. These are important considerations as the United Kingdom negotiates a trade deal with the United States in haste, whilst at the same time attempting to manage the ongoing coronavirus disease 2019 (COVID-19) pandemic. In this commentary, I reflect on changing attention to trade and nutrition during the COVID-19 pandemic in light of Townsend and colleagues’ analysis. I explore scope for greater attention to nutrition in US-UK trade negotiations, and the challenges created by the vested interests of major UK and US processed food exporters. I further discuss the utility of the theoretical tools employed by Townsend and colleagues for wider debates in the political economy of health.

## Health and Economic Policy: Attention Deficits

 For decades, researchers studying the health impacts of diverse policies, including fiscal retrenchment, welfare reform, and free trade agreements (FTAs), have demonstrated that policies that are expected to deliver economic gains sometimes run the risk of yielding significant harms to health, and may even fail to deliver on their economic promises too.^1-4^ What does it take for those involved in policy-making to recognize and mitigate these potential harms to health? Any attempt to answer this question will inevitably provoke discussion about politics, with debate about the relative importance of diverse and complex social and political factors. There is, however, one answer that will be obvious to many: evidence alone is not enough.

 Research on FTAs and health, for example, has consistently identified how reducing barriers to international commerce can usher in a suite of changes to the social and environmental determinants of health. To be sure, such changes are not universally inimical, as when, for example, economies that liberalised and experienced subsequent macro-economic gains also saw reductions in rates of child mortality or increased access to stable food supplies.^5,6^ Furthermore, researchers assessing the causal impacts of FTAs on health determinants and outcomes have yet to fully scrutinise the wide range of outcomes that may be affected.

 However, there is research to demonstrate select health risks from FTAs and related ‘trade liberalization’ policies that is relatively robust. In the absence of feasible randomized experimentation, studies assessing the causal effect of trade policies on health must necessarily rely on ‘natural experiment’ methods. These exploit quasi-random instances of policy change to identify impact, for example by comparing a country or countries exposed to a trade policy change to a comparable ‘counterfactual’ or ‘control group’ of countries that were not. For example, quasi-experimental analyses have consistently illustrated how trade agreements with the United States have contributed to a surplus of calories in food environments and increased the availability of calorie dense, ultra-processed foods, and sugar sweetened soft-drinks.^7-12^

 Descriptive analyses lend credence to these studies by illustrating the mechanisms through which trade deals modify food environments and ultimately lead to dietary change, including, for example, the role of increased investment by trans-national corporations in local food systems and heavy marketing of processed foods.^13-15^ Other studies have identified the pathways through which trade policy can impact on access to medicines, alcohol sales, and health policy space.^16-18^ Some will question the causal conclusions from descriptive accounts. However, both quasi-experimental and descriptive studies provide reasonable grounds to pause and question whether and how FTAs under discussion might yield health risks, and what action could be taken to mitigate avoidable harms.

 Those who have worked to establish these links have long lamented the lack of attention to these pathways to health impact, and the need for greater public health voice in trade policy discussions. Yet, such accounts often stop short of interrogating the underlying factors that limit attention to health concerns. Indeed, to do so requires a rather different set of theoretical and methodological tools to those used to interrogate the health impacts of trade.

 It is within this context that Townsend and colleagues provided important insight, using the tools of political science to unpack the processes that influenced whether those engaged in the Comprehensive and Progressive Agreement for Trans-Pacific Partnership (CP-TPP, formerly known as the TPP) acknowledged – and sought to mitigate – potential health challenges from the deal.^19^ The authors applied Shiffman and Smith’s framework of political prioritisation to analyse 25 interviews with relevant trade- and health-policy stakeholders.^20^ Their analysis revealed 16 dominant themes that featured in the interviews, including exporter interest or disinterest in expanding sales of unhealthy commodities, path-dependency in trade-policy-making, and public support for the health issues. The authors illustrated how these factors, among others, fostered political attention to the potential impact of the (CP-)TPP on access to medicines and tobacco policy space, whereas their absence may explain why attention to nutrition and alcohol was less prominent.

## Exogenous Events, Trade, and Health

 Perhaps one of the most intriguing findings identified by Townsend and colleagues concerns the role of ‘exogenous events’ in shaping awareness of the (CP-)TPP’s prospective impact on health policy. During (CP-)TPP negotiations, tobacco firm Phillip Morris litigated against the Australian government over its tobacco plain packaging legislation. This ‘exogenous event’ drew public attention to the potential risks of litigation for public health in the realm of tobacco, by showing how businesses can cite investor protections in trade deals to dispute tobacco control policies.

 To describe coronavirus disease 2019 (COVID-19) as an ‘exogenous event’ may read as an understatement of the human suffering and economic chaos wrought by the pandemic, but it is arguably a candidate for what Townsend et al refer to under this label. Notably, COVID-19 has brought the need to balance economic and health issues front and centre of political debates globally. In this context, many countries worldwide are currently negotiating new FTAs. Will COVID-19 prompt a shift in global discourse on trade, with greater attention to the health consequences of FTAs?

 The United Kingdom provides one relevant example to illustrate how these dynamics are starting to play out. The United Kingdom is currently seeking to negotiate a new trade deal with the US in haste following its departure from the European Union. UK public discourse on the health impacts of a US-UK deal has hitherto been dominated by concerns about the privatization of the NHS and deteriorating food standards, including importation of ‘chlorinated chicken.’^21^ Yet, policies to address poor nutrition have recently ascended the government’s agenda after the UK Prime Minister experienced complications with COVID-19 treatment due to being overweight.^22^ This raises the question as to whether increased public and political attention to nutrition policy as a result of COVID-19 could in turn prompt increasing to the implications of a UK-US FTA for nutrition and policy space.

 The first step is to connect the dots. Following Johnson’s successful recovery from COVID-19, the UK government has launched a new obesity strategy. But it could be ‘on the table’ in a US-UK trade deal. Changes to nutrition policy can happen during negotiations, as governments seek to reduce or harmonize regulations that affect trade, or afterwards, when businesses cite trade rule violations or expropriation of their investments to litigate against obesity prevention measures that have a detrimental impact on their exports, sales, and profits. For example, the UK government has proposed interpretative front-of-pack nutrition labelling to promote healthier diets, but US trade officials – under pressure from industry – have previously challenged trade partners’ attempts introduce such measures during trade negotiations and debates.^23,24^

 Other proposed obesity prevention measures could also be contested. For example, a proposed TV junk-food advertising ban could be said to limit trade in advertising services. If the United Kingdom ratifies an FTA similar to the United States–Mexico–Canada Agreement or CP-TPP (as seems likely), US businesses could threaten litigation if the United Kingdom adopts such a measure in future, leading the policy to be abandoned or watered down.^25^ US businesses could also claim a seat at the table in policy discussions over these and other policies, as regulatory transparency clauses in recent FTAs grant foreign stakeholders scope for input.

 Having committed to a suite of obesity prevention measures, leaving them open to US contestation during trade negotiations now represents a series of political risks for the Johnson government. One risk is that the government may simply be unable to follow through with its proposed policies, leading to an implementation failure. The second is reputational, as the government has publicly committed to a new obesity strategy. Agreeing to an FTA which signs away the UK’s scope to introduce these measures would be inconsistent with the government’s commitments or worse, it could look as though the government is recanting a prior policy commitment.

 Townsend and colleagues’ analysis nevertheless illustrates how myriad political factors shaped attention to health in Australia, and each of these interacting and potentially counter-balancing forces might come into play in the United Kingdom too. Notably, exporter interests are another key factor identified by Townsend and colleagues. The UK food and drink industry has much to gain from a possible trade deal; this includes Scottish whisky exporters, who currently face a 25% tariff on US imports of their products that was imposed following a dispute about subsidies awarded to Airbus.

 Industry has a long history of exploiting moments of crisis to push for liberalization reforms that expand their sales and profits.^26^ Indeed, the US government has already opposed Mexico’s front-of-pack labelling policies at World Trade Organization, citing a need to avoid regulation in the food sector in order to enable food producers to survive the uncertainties and costs wrought by the pandemic.^27^ Such arguments may find favour with the ideology of British ministers, many of whom have historically supported limited regulation as a means to stimulate the economy.

 In short, COVID-19 has certainly generated increased awareness to the importance of health to the economy and, in the UK context at least, the need for further interventions to address poor nutrition and obesity. It is, however, also the case that vested interests and their ideological foundations may serve as a powerful counterpoise that jeopardizes the UK’s new health policy ambitions. Townsend and colleagues’ innovative analysis provides a framework for identifying how these counter-acting factors can play out. As such, their insights are likely to be informative for scholars and policy-makers interested in the UK’s trade negotiation processes, and those seeking to mitigate any health harms from the UK’s future deals. A failure to draw on such insights and to recognize these political pressures could squander a window of opportunity for health.

 How the UK situation evolves, and whether and how COVID-19 influences attention to health in FTA negotiations, will also provide insights for elsewhere. We shall ultimately learn whether and how lessons from the pandemic are brought into consideration in FTA discussions and how prominently they are considered in comparison to competing demands. This could prove relevant more broadly by identifying the extent to which framing trade and health issues in relation what has been learnt from the pandemic could help or hinder those seeking to raise attention to health issues in trade negotiations in other contexts. The insights may prove particularly relevant for developing countries which typically have a weaker hand in trade negotiations with powerful, economic partners, and may alternatively have to rely on powerful frames and ideas to secure health protections.

## The Political Economy of Health: From “What Happens?” to “Why?”

 Regardless of the UK’s fate in its negotiations for a US deal, Townsend and colleagues rightfully observe that research on the social and commercial determinants of health more broadly could fruitfully benefit from greater application of political prioritisation frameworks. In the realm of economic policy, research in the ‘political economy of health’ has identified how diverse economic reforms shape the determinants of health, both for better and for worse, including fiscal policy reform, welfare retrenchment, and labour de-regulation.^28-30^

 As research accumulates, there is much to benefit from the application of political process frameworks for studies on these topics too. As Basu and colleagues noted in their review of research on austerity and health, for example, studies have convincingly demonstrated how “the cutting of social support systems results in horrendous ruin, morbidity and death for many people.”^31^ McCartney and colleagues similarly concluded a review of political economy of health scholarship by noting that “neoliberal restructuring seems to be associated with increased health inequalities”^32^; this included, but is not limited, to trade and austerity scholarship.

 For scholars investigating these and other topics in the political economy and health, Townsend and colleagues make a compelling case for further research using political science frameworks to go beyond asking ‘what is the effect of economic policy on health?’ of to asking a logical, albeit challenging, follow-up: ‘why aren’t adverse effects being recognized and adequately mitigated?’

## Ethical issues

 Not applicable.

## Competing interests

 Author declares that she has no competing interests.

## Author’s contribution

 PB is the single author of the paper.
